# Harnessing computational tools of the digital era for enhanced infection control

**DOI:** 10.1186/s12911-024-02650-9

**Published:** 2024-09-12

**Authors:** Francesco Branda

**Affiliations:** Unit of Medical Statistics and Molecular Epidemiology, Università Campus Bio-Medico di Roma, Rome, Italy

## Abstract

This paper explores the potential of artificial intelligence, machine learning, and big data analytics in revolutionizing infection control. It addresses the challenges and innovative approaches in combating infectious diseases and antimicrobial resistance, emphasizing the critical role of interdisciplinary collaboration, ethical data practices, and integration of advanced computational tools in modern healthcare.

## Main text

The modern era is characterized by an explosion of computational innovations that are revolutionizing every aspect of our lives, including healthcare. In the face of the growing challenges posed by infectious diseases and antimicrobial resistance, it is critical to take full advantage of the transformational potential of computational tools to reform and improve infection control strategies. Infectious diseases place a significant burden on healthcare systems around the world. Despite medical advances, we are continually engaged in a battle against the emergence of new pathogens and the rapid spread of already known infectious agents. In addition, growing antimicrobial resistance threatens to render some of our most valuable treatments ineffective. These challenges require an innovative and enhanced approach to surveillance, prevention, and infection control.

In this context, computational tools such as Artificial Intelligence (AI), Machine Learning (ML) and Big Data Analytics (BDA) are emerging as crucial allies. AI refers to systems that simulate human intelligence to perform tasks and can improve autonomously based on data. In recent years, advances in natural language processing (NLP) have facilitated the extraction of valuable information from large amounts of unstructured data, such as electronic medical records and clinical research data [1, 2]. Major innovations in this field include large language models (LLMs) and multimodal models (MLLMs) [3]. The former can analyze and generate text in natural languages, while the latter combine text, images, and other types of data to provide richer and more complex analyses. For example, AI can be used to detect infectious disease outbreaks early by analyzing data from sources such as surveillance reports and social media, thus providing early warning systems that enable timely and targeted responses [4]. Moreover, AI chatbots such as ChatGPT, are increasingly being used to assist public health practitioners in co-designing mathematical transmission models, enhancing strategies for infection control and outbreak management [5]. ML is a sub-discipline of AI that focuses on using algorithms to analyze data and make predictions or decisions based on it. For example, ML can be used to analyze antimicrobial prescribing patterns and identify optimal practices, thus helping to slow the development of antimicrobial resistance. These tools can also be used to tailor treatment regimens to patient and pathogen characteristics, maximizing treatment efficacy and reducing the risk of resistance [6, 7]. Studies have shown that ML models can predict antibiotic resistance based on patient data and local microbial trends, leading to more personalized and effective treatments [8]. BDA refers to the use of advanced techniques to collect, process, and analyze large volumes of data from a variety of sources. By combining different types of data, BDA enables the development of more accurate predictive models and a better understanding of the mechanisms underlying the spread of infections and the emergence of antimicrobial resistance. A significant example is the use of genomic data with epidemiological information, which has enabled researchers to trace the origins and transmission routes of various pathogens, providing crucial information for epidemic control [9]. The volume of Internet of Things (IoT)-generated data is considered a major source of big data and in healthcare also offers promising opportunities for infection control [10]. IoT devices can continuously monitor patient health parameters, environmental conditions, and equipment status, providing real-time data that can be analyzed to detect early signs of infection and prevent the spread of diseases within healthcare facilities [11]. Additionally, blockchain technology holds potential for improving data security and integrity in infection control efforts. By providing a decentralized and tamper-proof ledger for health data, blockchain can ensure the accuracy and trustworthiness of data used in computational analyses and decision-making processes [12]. In summary, while AI and ML provide predictive analytics and pattern recognition capabilities, BDA provides the necessary context for understanding and interpreting such analytics on a large scale.

Despite considerable potential, the implementation and evaluation of these computational methods in infection control present significant challenges that deserve careful consideration. A major problem is clinical validation and generalizability. Many AI and ML models are developed and validated on specific datasets, often from individual institutions, which limits their applicability in different clinical settings and populations [13]. This lack of generalizability can lead to models that work well in one setting but fail in another, with the risk of suboptimal results for different patient groups [14]. The issue of interpretability and transparency is equally crucial. Advanced models, particularly those based on deep neural networks, often operate as “black boxes” [15, 16], making it difficult for clinicians to understand the reasoning behind their predictions or recommendations. In infection control, where decisions can have vital consequences, the ability to interpret these models is critical to building trust and ensuring widespread adoption. Clinicians must be able to understand and explain how these tools arrive at their conclusions, especially in high-risk scenarios. However, a significant challenge is the gap between healthcare professionals and experts in information technology and data analytics. To fully harness the power of these tools, we need to foster multidisciplinary collaborations and improve mutual understanding between these fields. Bringing together clinicians, data scientists, and computational experts is essential to successfully integrate these technologies into healthcare [17]. Bias and equity are additional concerns arising from the fragmented and incomplete nature of health data. AI and ML models trained on biased data may unintentionally perpetuate or even exacerbate existing disparities in healthcare [18, 19]. For example, models that do not account for socioeconomic or racial differences may produce skewed outcomes, potentially leading to inequitable treatments for different groups of patients. This is of particular concern in infection control, where health disparities could worsen if these biases are not adequately addressed during the development and dissemination of these models. Another challenge is ensuring data quality and representativeness. Infection control activities often rely on data from a variety of sources, including electronic health records [20], surveillance systems and laboratory data [21]. However, these sources often have inconsistencies, incompleteness or structural inadequacy, complicating their use in computational analysis for the development of reliable models. Integrating these advanced tools into existing clinical workflows introduces significant organizational challenges, including the need for alignment between technology and healthcare practices, which is critical for effective implementation [22, 23]. Finally, it is important to consider ethical and regulatory aspects. The use of AI and ML in infection control raises important questions about data privacy, consent, and potential misuse of sensitive health information [24, 25]. Robust data governance frameworks are needed to ensure the protection of patient privacy while enabling meaningful research and innovation. These frameworks must strike a delicate balance between facilitating the advancement of AI and ML technologies and protecting individual rights.

The convergence of computational science and infection control represents an exciting and promising frontier for public health. As we move toward a future in which advanced digital tools become an integral part of our prevention and control strategies, it is necessary to maintain a balanced approach. Technological innovation must go hand in hand with ethical considerations, interdisciplinary collaboration, and an ongoing commitment to improving the quality and accessibility of health data. Only through this holistic approach can we hope to take full advantage of the transformative potential of these technologies, creating more responsive, effective, and resilient infection control systems. Success in this area will not only improve our ability to address current challenges, but also enable us to anticipate and mitigate future threats to global health. As we advance into this new era of technology-enhanced infection control, let us remember that our ultimate goal remains unchanged: to protect and improve human health. Computational innovations are powerful tools in this mission, but it is the wisdom, empathy and dedication of healthcare workers that will continue to drive progress in this crucial field.

## Data Availability

No datasets were generated or analysed during the current study.

## Abbreviations

AI: Artificial Intelligence
ML: Machine Learning
COVID-19: Coronavirus disease 2019
NLP: Natural Language Processing
IoT: Internet of Things
